# Effects of child characteristics and dental history on dental fear: cross-sectional study

**DOI:** 10.1186/s12903-018-0496-4

**Published:** 2018-03-07

**Authors:** Mohammad A. Alshoraim, Azza A. El-Housseiny, Najat M. Farsi, Osama M. Felemban, Najlaa M. Alamoudi, Amani A. Alandejani

**Affiliations:** 1 Ministry of Health, Jeddah, Saudi Arabia; 2 Paediatric Dentistry, Faculty of Dentistry, King Abdulaziz University, P.O. Box: 80200, Jeddah, 21589 Saudi Arabia; 3 Paediatric Dentistry, Faculty of Dentistry, Alexandria University, Alexandria, Egypt; 4 National Guard Hospital, King Abdulaziz Medical City, Jeddah, Saudi Arabia

**Keywords:** Dental fear, Dental anxiety, Children’s fear survey schedule-dental subscale (CFSS-DS), Caries, Dental behaviour

## Abstract

**Background:**

Dental fear (DF) is a challenging problem in dentistry. It is multifactorial in origin and many contributing factors have been identified. The aim of the study was to assess dental fear among 12–15 years old Arabic speaking children in Jeddah, Saudi Arabia and its relation to demographic variables, previous dental experience, and child behaviour.

**Methods:**

In this cross-sectional study, a total of 1522 boys and girls from middle schools in Jeddah, Saudi Arabia participated in this study during the period of 2014 to 2016. The Children’s Fear Survey Schedule–Dental Subscale (CFSS-DS) was used to assess DF. A parental questionnaire was used to record the children’s previous dental experience. Children were examined for caries and the children’s behaviour was assessed during dental examination using Frankl Behaviour Rating Scale. The associations between different variables and the CFSS-DS scores were analysed using t-tests, ANOVA, and multiple linear regression analysis.

**Results:**

The response rate of the questionnaires was 78.6%. The mean CFSS-DS score was 25.99 ± 9.3 out of a maximum of 75. Bivariate analysis showed that younger children, girls, and public-school students were significantly more fearful than older children, boys, and private school children, respectively (*P* < 0.001). Children who showed poor behaviour during dental examination were significantly more fearful than those with good behaviour (*P* < 0.001). Regression analysis showed that children who had significantly higher scores of dental fear were the children who did not visit the dentist in the past year due to dental fear; who never visited the dentist or those who only visited the dentist on pain; who were reported by parents as crying, screaming, or resistant during their previous dental visit; and those who were described to be in pain during previous dental treatment. Dental caries showed no significant association with DF.

**Conclusions:**

This study confirms that DF is low among 12–15 years old Arabic speaking children in Jeddah, Saudi Arabia. DF is associated with age, gender, school type, irregular patterns of dental visits, painful experiences during previous dental visits and negative behaviours during dental examinations.

**Electronic supplementary material:**

The online version of this article (10.1186/s12903-018-0496-4) contains supplementary material, which is available to authorized users.

## Background

Dental fear (DF) is a widely extended physiological, behavioural, and emotional reaction to one or more threatening stimuli in the dental practice [1]. DF and dental anxiety (DA) are terms that are often mixed up in the literature; thus, dental fear and anxiety (DFA) is used to describe all kinds of fear and anxiety related to dentistry [1]. The use of self-report scales is the most common and reliable method of measuring DFA, and the Children’s Fear Survey Schedule-Dental Subscale (CFSS-DS) is one of the most commonly used scales [2]. This scale has been validated in different populations with different languages [2].

DF is multifactorial in origin and many factors that affect it have been identified [1]. One of these factors is the age of the child, which has been associated with DF scores, although this is a matter of debate. One previous study reported that there was no effect of age on DF [3], while other studies found increased DF in older children compared to younger ones [4, 5]. DF cannot be considered to be stable over time since other factors may decrease DF with age such as treatment variables and subjective experience [6]. The sex of the child also has a significant effect on DF severity [7]. Some studies showed significant differences in DFA between the two sexes [7–9], while other studies found no significant differences [10–14].

Factors related to dental history, such as caries experience, previous dental visits, dental visit patterns, type of previous dental treatments, and behaviour during dental treatment, and their relationship with DF have been discussed in previous studies [6, 13, 15, 16]. It has been suggested that prior dental visits can decrease DF since this can eliminate negative thoughts about dentistry [15]. However, the type of dental treatment the children received in their previous dental visits plays a significant role in DF severity [6].

Among population-based studies in children, there is still debate regarding the relationship between DF and previous dental history. Thus, further investigation into factors associated with DF is needed. The aim of the study was to assess dental fear among 12–15 years old Arabic speaking children in Jeddah, Saudi Arabia and its relation to demographic variables, previous dental experience, and child behaviour.

## Methods

### Study design

This is a cross-sectional study and the guidelines of Strengthening the Reporting of Observational Studies in Epidemiology (STROBE) were followed in reporting this study [17].

### Participants

The sample consisted of 1522 middle school-aged children who were selected randomly from schools in Jeddah, Saudi Arabia. A consent form was sent to parents, and the inclusion criteria were as follows: aged between 12 and 15 years, native language was Arabic, and signed informed consent. The population frame included all children registered in middle schools in Jeddah according to Ministry of Education, which included 115,689 children attending public or private middle schools. The prevalence of DF in the target population was hypothesized to be 20% according to previous population based studies [1], so the percentage frequency of the outcome factor was set at 20% with ±2% confidence limits. The confidence level was set at 95%, the significance level was set at 5%, and the power was set at 85%. The resulting needed sample size for the study was 1520 subjects.

The sampling method utilized a multistage stratified random sample according to the four districts of Jeddah, gender (male and female schools), and then according to school type (private and public). Four schools were chosen from each district: a male public school, a male private school, a female public school, and a female private school. The sampling procedure yielded a total of 16 school representative of the total number of schools in Jeddah. For each school, one class from each grade was randomly assigned (using the bowl method) to join the study. Where there was a small number of students in any selected class (less than 15), another class was randomly selected. Where there was a small number of students in the school (less than 150), all students in the school were included in the study.

Ethical approval was received from the Research Ethics Committee, Faculty of Dentistry, King Abdulaziz University, Jeddah, Saudi Arabia (number: 046–15).

### The questionnaire

The study variables were assessed using two questionnaires. The first questionnaire was for the parents and included the consent form as well as questions to investigate the different factors affecting DF. The factors included: pattern of previous dental exposure, and the child behaviour during those visits. The parents’ questionnaire (Additional file 1) was adopted from a previous study questionnaire [18] developed based on the literature. In this study, the selected questions were revised by four experts in paediatric dentistry interested in behaviour management of children. The second questionnaire was the Arabic version of the CFSS-DS [19], which was completed by children in order to assess their DF level. The Arabic version of the CFSS-DS is highly reliable in terms of both test-retest reliability and internal consistency and shows good criterion validity and moderate construct validity [20, 21]. It has the same 15 items of the English version and each item was scored on a five Likert scale ranging from 1 to 5. Higher scores indicated higher dental fear. The scores of the 15 items were added to get a total score for each child.

### Study procedures

Multiple school visits were carried out during this study. The study started in September 2014 and ended in June 2016. During the first visit, the questionnaires with consent forms were distributed to the selected children. Subsequently, the examiners visited the schools again over a five-month period for data collection. The examiners collected the parent’s questionnaires from students. and only those with parental approval (i.e. signed informed consent form) moved on to the second phase of the study, i.e. the child’s questionnaire. The questionnaires were self-reported and filled out directly by the children during school hours. On collecting the child’s questionnaire, the questions were inspected for completion for each child. In case of any missing data, the child was asked to complete the questionnaire. After completing the child’s questionnaire, a dental examination was carried out to assess the children caries experience. The examination was carried out using the Community Periodontal Index (CPI) probe (Screen Probe Shepherd’s Hook 11.5 d722 pcwho-23, lot/ 981,766, Nordent, USA) and a mirror using adequate lighting and infection control measures. The index for decayed, missing, or filled permanent teeth (DMFT) was recorded according to the World Health Organisation (WHO) criteria [22, 23]. Dental caries severity was categorized based on a modification of the WHO criteria for dental caries severity into the following: no caries (DMFT = 0), low caries (DMFT< 2.7), moderate caries (DMFT = 2.7–4.4), and severe dental caries (DMFT≥4.5) [23]. The examination was carried out by two trained and calibrated examiners. The kappa value for inter-examiner reliability was 0.93 and for the intra-examiner reliability ranged from 0.8 to 1.00.

During the dental examination, the children’s behaviour was evaluated according to the Frankl Behaviour Rating Scale [24] by two calibrated investigators to assess the relationship between DF and child behaviour. The classification was dichotomized into two categories due to the low numbers in some categories. Negative behaviours included the two categories of definitely negative and negative behaviours, while positive behaviour included the two categories of positive and definitely positive behaviours. Kappa statistic was calculated and its value for inter-examiner reliability was 0.93 and it ranged from 0.83 to 1.00 for the intra-examiner reliability. After the dental examination and behavioural assessment, a confidential report was sent to parents to inform them about their child’s dental status and to advise them to visit a dentist if needed.

### Data analysis

Statistical analyses were carried out using SPSS version 18.0 (SPSS Inc., Chicago, II, USA). The significance level was set at *P* < 0.05. For each child, the total fear score was calculated by adding the fear score of each of the 15 items of the self-reported CFSS-DS, which ranged from 15 to 75, with higher scores indicating higher fear levels.

The associations between dental fear and demographic variables, previous dental experience, caries experience, and behaviour were analysed using independent t-tests and one-way analysis of variances (ANOVA). When significant effects were found using a one-way ANOVA, a Tukey post-hoc test was used to determine significant intergroup mean differences. A multiple linear regression analysis was used to evaluate significant predictors of dental fear levels while controlling for potential confounders.

## Results

The consent form and parent questionnaires were distributed to 2000 children, out of which 1572 children returned the consent form and the parent questionnaire. Thus, the response rate for all children was 78.6%. Of those who returned the consent form, 1522 participated in the study (19 children refused to participate in the study, and 31 children were absent on the examination days).

There were 826 (54.27%) male participants and 696 (45.73%) female participants. There were 1027 (67.5%) children from public schools and 495 (32.5%) children from private schools. All children were aged between 12 and 15 years, with a mean age of 13.5 ± 1.05 years.

The results in Table 1 show that the DF scores were statistically significantly different among the different age groups (*P* < 0.001). A post-hoc analysis indicated that 12-year-old children had statistically significantly increased DF scores compared to other age groups. The mean DF score in girls was significantly higher than that in boys (*P* < 0.001). The mean DF score was also significantly higher in children attending public schools compared to children attending private schools (*P* < 0.001). Table 2 shows the scores for each item in the CFSS-DS of the participants. The mean DF total score (CFSS-DS) for all participants was 25.99 ± 9.31.

**Table 1:** Mean dental fear in relation to age, sex, and school type

Variables		N (%)	Mean (SD)	95% CI		Min–Max	Test value (*P* value)
				Lower Bound	Upper Bound		
Age	12	312 (20.5)	27.96 (11.23)	26.71	29.21	15–74	F = 7.42^‡^ (< 0.001*)
	13	464 (30.5)	26.01(9.07^a^)	25.18	26.84	15–63	
	14	413 (27.1)	25.55 (8.47^a^)	24.73	26.37	15–65	
	15	333 (21.9)	24.63 (8.35^a^)	23.73	25.53	15–68	
Sex	Male	826 (54.3)	23.12 (7.12)	22.63	23.60	15–68	*t* = 193.16^†^ (< 0.001*)
	Female	696 (45.7)	29.39 (10.41)	28.62	30.17	15–74	
School Type	Public	1027 (67.5)	26.52 (9.75)	25.93	27.12	15–74	*t* = 10.55^†^ (< 0.001*)
	Private	495 (32.5)	24.87 (8.23)	24.14	25.60	15–69	
Total		1522 (100)	25.99 (9.31)	25.52	26.45	15–74	

*N* total number of children, *SD* Standard deviation, *CI* Confidence interval

*Statistically significant (*P* < 0.05), ^‡^Analysis of variance, ^†^ t-test

Means sharing the same alphabetical letter superscripts are not significantly different from each other (post-hoc, *P* ≥ 0.05)

Means that have different alphabetical letter superscripts are significantly different from each other (post-hoc, *P* < 0.05)

**Table 2:** CDSS-DS mean item scores for all children, boys and girls

Item	Total (*N* = 1522)	
	Mean (SD)	95% CI
1.Dentists	1.61 (0.89)	1.5–1.6
2.Doctors	1.41 (0.76)	1.3–1.4
3.Injection	2.21 (1.22)	2.1–2.2
4.Somebody examining your mouth	1.38 (0.75)	1.3–1.4
5.Having to open your mouth	1.43 (0.78)	1.3–1.4
6.Having a stranger touch you	1.81 (1.07)	1.7–1.8
7.Having somebody look at you	1.43 (0.80)	1.3–1.4
8.The dentist drilling	2.42 (1.22)	2.3–2.4
9.The sight of the dentist drilling	2.16 (1.19)	2.1–2.2
10.The noise of the dentist drilling	2.04 (1.14)	1.9–2.1
11.Instruments in your mouth	1.93 (1.08)	1.8–1.9
12.Choking	2.29 (1.21)	2.2–2.3
13.Having to go to hospital	1.34 (0.75)	1.3–1.3
14.People in white uniforms	1.14 (0.52)	1.1–1.1
15.Dentist cleaning your teeth	1.39 (0.76)	1.3–1.4
Total	25.99 (9.31)	25.5–26.4

*CFSS-DS* children’s fear survey schedule–dental subscale, *N* total number of children, *SD* standard deviation, *CI* confidence interval

Regarding previous dental exposure, Table 3 shows that 1340 (88%) of the parents reported that their children had previous dental experience, while only 182 (12%) of the children had no previous dental experience. There was no significant difference in the mean DF scores between children with previous dental experience and those without previous dental experience (*P* = 0.230), or between those who visited the dentist last year and those who did not (*P* = 0.931). The reasons for not visiting the dentist last year included money issues, no pain, no time for treatment, not needed, and child fear. A post-hoc analysis indicated that children who did not visit the dentist due to “child fear” had the highest DF scores compared to other causes (*P* < 0.001). Parents were asked about their children’s dental visit patterns, and the results show that the DF scores differ significantly among the different patterns of dental visit (*P* < 0.001). Children who visit the dentist only when they have pain show significantly increased DF scores compared to those who visit the dentist on a regular basis (*P* < 0.001).

**Table 3:** Mean dental fear scores in relation to dental history

Dental history variables		N (%)	Mean (SD)	95% CI	Test value (*P* value)
Previous dental exposure	Yes	1340 (88)	25.88 (9.1)	25.4–26.4	1.441^†^ (0.230)
	No	182 (12)	26.76 (10.6)	25.2–28.3	
Dental visit in previous year	Yes	843 (55.4)	26.00 (9.1)	25.4–26.6	0.007^†^ (0.931)
	No	679 (44.6)	25.96 (9.6)	25.2–26.7	
Why didn’t your child visit dentist last year?	Money	56 (4.3)	26.30 (9.8^a^)	23.7–28.9	7.53‡ (< 0.001*)
	No pain	410 (26.9)	25.58 (9.1^a^)	24.7–26.5	
	No time	48 (3.2)	25.33 (9.2^a^)	22.6–28.0	
	Not needed	187 (12.3)	25.01 (8.1^a^)	23.8–26.2	
	Child fear	52 (3.4)	36.33 (14.2)	32.4–40.3	
Frequency of dental visits	Regular	130 (8.5)	24.34 (6.7^ac^)	23.2–25.5	7.161^‡^ (< 0.001*)
	With pain	1014(66.6)	26.76 (10.8^b^)	25.9–27.1	
	Sometimes	196 (12.9)	23.62 (6.6^c^)	22.7–24.5	
	Never	182 (12)	26.51 (9.7^ab^)	25.2–28.3	
Previous dental treatment	Examination	617 (40.5)	25.50 ± 8.8	24.8–26.2	1.52^‡^ (0.179)
	Anaesthesia	214 (14.1)	25.69 ± 8.3	24.6–26.8	
	Extraction	527 (34.6)	26.56 ± 9.7	25.7–27.4	
	Filling	659 (43.3)	25.56 ± 9.1	24.9–26.3	
	Prophylaxis	225 (14.8)	26.48 ± 10.1	25.1–27.8	
	Others	89 (6.4)	24.56 ± 7.7	22.9–26.1	
Child behaviour during previous visit/s§	Crying	90 (5.9)	34.51 (11.3^a^)	32.1–36.9	32.5^‡^ (< 0.001*)
	Screaming	49 (3.2)	30.29 (12.3^ab^)	26.8–33.1	
	Resistant	373 (24.5)	28.00 (9.7^bc^)	27.0–29.0	
	Cooperative	738 (48.5)	24.12 (7.8^d^)	23.6–24.7	
	Happy	122 (8%)	22.83 (7.0^d^)	21.6–24.1	
	I don’t know	135 (8.9)	26.07 (9.9^dc^)	24.4–27.8	
Pain during previous dental treatment/s§	Yes	456 (30)	28.82 (10.87^a^)	27.8–29.8	38.20^‡^ (< 0.001*)
	No	420 (27.6)	23.54 (7.97^b^)	22.8–24.3	
	A little	631 (41.5)	25.53 (8.35^c^)	24.9–26.1	

*N* total number of children, *SD* standard deviation, *CI* confidence interval

*Statistically significant (*P* < 0.05), ^‡^Analysis of variance, ^†^ t-test

Means sharing the same alphabetical letter superscripts are not significantly different from each other (post-hoc, *P* ≥ 0.05)

Means that have different alphabetical letter superscripts are significantly different from each other (post-hoc, *P* < 0.05)

^§^15 (1%) of parents did not answer the question regarding the behaviour of their children

Regarding previous dental treatment, Table 3 shows a non-significant difference in DF scores among children who have experienced different dental treatments (oral examination, local anaesthesia, extraction, filling, prophylaxis, and other) (*P* = 0.179). Children who cried during their previous dental visits were significantly more fearful compared to those who displayed other behaviours (*P* < 0.001), except for screaming. Children who felt pain during previous dental treatment had significantly higher mean DF scores compared to those who did not feel any pain or those who felt little pain (*P* < 0.001).

Upon dental examination, 232 (15.2%) children had “no caries,” 330 (21.7%) had “low caries,” 418 (27.5%) children had “moderate caries,” and 542 (35.6%) children had severe dental caries. For the total sample, children with severe dental caries had the lowest mean DF score. This score was significantly lower than that for children who had no caries or who were classified as having low caries (*P* < 0.001; Table 4).

**Table 4:** Mean dental fear scores in relation to caries experience in permanent teeth

Caries severity	Total			F Value (*P* value)
	N (%)	Mean (SD)	95% CI	
No caries	232 (15.2)	27.91 (10.4^a^)	26.6–29.3	10.23‡ (< 0.001*)
Low caries	330 (21.7)	27.36 (9.3^ab^)	26.3–28.4	
Moderate caries	418 (27.5)	25.96 (9.3^bc^)	25.1–26.9	
Severe caries	542 (35.6)	24.51 (8.6^c^)	23.8–25.2	
Total	1522 (100)	26.05 (9.3)	25.6–26.5	

*N* total number of children, *SD* standard deviation, *CI* confidence interval

*Statistically significant (*P* < 0.05), ^‡^Analysis of variance

Means sharing the same alphabetical letter superscripts are not significantly different from each other (post-hoc, *P* ≥ 0.05)

Means that have different alphabetical letter superscripts are significantly different from each other (post-hoc, *P* < 0.05)

No caries DMFT = zero, low caries < 2.7, moderate caries ≥2.7 to ≤4.4, severe caries≥4.5

Table 5 indicates that the children displaying negative behaviours during the dental examination had significantly higher mean DF scores compared to the children displaying positive behaviours (*P* < 0.001).

**Table 5:** Mean dental fear scores in relation to behaviour during dental examination

Behaviour	N (%)	Mean (SD)	95% CI	Test value (*P* value)
Negative	517 (34)	29.37 (10.5)	28.4–30.3	110.39^†^ (< 0.001*)
Positive	1005(66)	24.23 (8.1)	23.7–24.7	
Total	1522 (100)	25.97 (9.3)	25.5–26.4	

*Statistically significant (*P* < 0.05), ^†^t-test

Negative behaviour includes definitely negative and negative behaviours

Positive behaviour includes positive and definitely positive behaviours

A multiple linear regression model was used to evaluate the associations of the variables while controlling for confounders (Table 6). The model showed that dental fear score decreases by 0.62 for every year increase in age after controlling for confounders. Females have a score of dental fear of 5.36 higher than males after controlling for confounders. Participants from private schools had a dental fear score of 1.92 less than participants from public schools. Children who did not visit the dentist last year due to child fear had a score of dental fear higher by 4.57 compared to children who did not visit the dentist last year because of no pain. Children who never visited a dentist or those who only visit the dentist on pain had a significantly higher dental fear score compared to children who visit the dentist regularly. Children who were crying, screaming, or resistant during their previous visit had significantly higher score of dental fear compared to children who were cooperative during their previous dental visit. Children who had pain during previous dental treatment had higher scores of dental fear compared to those who did not have pain in the previous treatment visit. Children whose behaviour was rated as negative during dental examination had higher dental fear score by 5.73 compared to those who were rated as having positive behaviour. Caries level was not associated with dental fear after controlling for confounders.

**Table 6:** Multiple linear regression model

Variable name	Beta (SE)	95% CI	*p*-value
Age	− 0.63 (0.22)	−1.06 – 0.20	0.004
Gender			
Female	5.36 (0.45)	4.48–6.24	< 0.001*
Male	ref	–	–
School			
Private	−1.923 (0.46)	− 2.83 – − 1.02	< 0.001*
Public	Ref	–	–
Why did you not visit the dentist last year			
Money	1.29 (1.17)	−0.100 – 3.59	0.269
No time	−0.31 (1.21)	− 2.70 – 2.07	0.799
No need	−0.80 (0.67)	− 2.12 – 0.52	0.238
Child fear	4.57 (1.24)	2.13–7.00	< 0.001*
No pain	Ref	–	–
Frequency of dental visits			
On pain	2.23 (0.73)	0.80–3.65	0.002*
Sometimes	0.30 (0.89)	−1.44 – 2.05	0.732
Never	4.45 (1.04)	2.40–6.50	< 0.001*
Regular	ref	–	–
Child behaviour during previous visit			
Crying	6.12 (1.00)	4.16–8.07	< 0.001*
Screaming	3.50 (1.23)	1.09–5.92	0.005*
Resistant	1.81 (0.54)	0.75–2.86	0.001*
Happy	− 0.88 (0.82)	−2.50 – 0.74	0.286
Do not know	1.40 (0.85)	−0.27 – 3.08	0.100
Cooperative	Ref	–	–
Pain during previous dental treatment			
Yes	3.04 (0.63)	1.82–4.27	< 0.001*
A little	1.13 (0.57)	0.02–2.24	0.046*
No	ref	–	–
DMFT			
Low caries	0.42 (0.75)	−1.06 – 1.90	0.578
Moderate caries	−0.03 (0.70)	−1.41 – 1.35	0.966
Severe caries	−0.03 (0.70)	−1.36 – 1.31	0.970
No caries	ref	–	
Behaviour during dental examination			
Negative	5.73 (2.1)	1.61–9.84	0.006*
Positive	ref		

*Statistically significant (*P* < 0.05),

## Discussion

This is an observational and analytical cross-sectional study designed to assess the severity of DF and associated factors in children aged 12–15 years. The mean DF score on the CFSS-DS for all children was 25.99 ± 9.31. This resembles the score reported in a recent study among children in Jeddah, Saudi Arabia [20]. This suggests a low level of DF amongst children in this age group, according to the cut-off score of 32 that was used in previous studies [3, 25, 26]. A straight forward comparison with other studies is difficult because of different study designs, sampling methods, different dental fear scales used (e.g. CFSS-DS, Corah’s Dental Anxiety Scale (CDAS), Modified Dental Anxiety Scale (MDAS), Short Dental Fear Questionnaire (SDFQ), Dental Fear Survey (DFS), Visual Analogue Scale (VAS), Facial Image Scale (FIS), Smiley Face Program (SFP), and Short Version of the Dental Anxiety Inventory (S-DAI)) and cultural and social factors [1]. However, the score is higher than the scores reported for Dutch children [3], and lower than the scores reported for US children [12], Japanese children [8], and Croatian children [27]. In addition, the difference in dental fear between children in Arabic speaking countries and developed countries may be related to differences in the organization of the dental health care systems [10]. While developed countries emphasize on prevention, a high number of children in this study only visit the dentist when they are in pain.

This study showed that the most feared items reported by the children were “dentist drilling,” “choking,” and “injection.” These items were found to be the most feared items among children with different rankings in different studies [8, 12, 19, 28]. The high scores on these items in different cultures indicates that children have the same concerns about specific dental procedures, even when the overall DF severity is different [8]. The use of these three items as screening tools was suggested by Cuthbert and Melamed who developed the CFSS-DS [12].

Our results show that girls are significantly more fearful than boys. Some studies support these findings [7–9, 20, 29], while others do not [10–12, 30]. It has been suggested that girls are more fearful of the dentist because of their tendency to show their feelings, unlike boys who may deny their fear [7, 27]. In addition, social and cultural factors were identified to play at least partial roles in this difference between fear levels in boys and girls [31]. For example, while in some cultures it is socially acceptable for girls to exhibit their fear, boys cannot [19]. Moreover, in some societies, such as Saudi Arabia, there is complete separation between boys and girls from an early school age, so they may not share the same social factors affecting DF. The separation between male and female peer groups that takes place at a late age in African populations was suggested to be at least a minor factor in this finding [31].

Our data demonstrate a decrease in DF scores in older children. These findings are supported by some previous studies [10, 12, 28, 29], while other studies do not support this [4, 5, 8]. In previous studies, the decrease in DFA that occurs over time could be related to the increase in general competence as children grow up, and maturation of cognitive controls and impulse control to improve the personality of the child [1]. The relationship between DF and age is not linear, as a weak negative correlation was found [12]. Previous studies have reported that age does not affect DF [8], no correlation [3, 21] or a weak correlation [20] between DF and age, or an increase in DF at an older age [5, 32]. Similarly, for 3–15-year-old Finnish children, higher DF scores were reported among 12–15-year-olds [4]. DF cannot be considered a stable factor with age since oral health status and exposure to different social and cultural events seem to affect the relationship between DF and age [6].

Our results show that 12% of the children in our study had never visited the dentist before. These children had higher DF scores compared to children with previous dental experience, although the difference was not significant. The association between DF and having never been to the dentist has been confirmed previously [15, 33, 34]. Children who had previously visited the dentist had low DA, and they were also more cooperative than children who had never visited the dentist. This is because children who have never visited the dentist usually have incorrect thoughts about dental procedures [15]. In the present study, about two-thirds of the children only visited the dentist owing to pain and their DF score was significantly higher than that for children who visit the dentist on a regular basis. This is in accordance with two longitudinal studies that found strong associations between irregular dental visits and DA [13, 16]. Both studies suggested that visiting the dentist in an irregular and symptomatic pattern is an indicator for DA [13, 16].

Children with a history of extraction had a higher score of dental fear compared to those without history of extraction. Children who had dental fillings were less fearful than children who had no history of dental fillings. The variation in the degree of invasiveness between different restorative techniques, as well as using atraumatic restorative methods, might explain the lack of association between DF and restorative treatment. However, most of the children received more than one treatment during their previous dental visits so the relationship between DF and any one specific dental treatment was difficult to observe. In general, it is recommended that children start with neutral dental visits (e.g. oral examination, and prophylaxis) before starting invasive treatment, since children who were exposed to invasive treatment during their first dental visit are more fearful [35].

Children whose parents reported them to be cooperative during their previous dental appointments were significantly less fearful compared to children who were crying or screaming, and even children who showed resistance behaviour during their previous dental visits. Crying during previous dental treatment was previously reported to be significantly positively related to DF [36]. Furthermore, children who showed uncooperative behaviour during the dental examination in the current study had a significantly higher mean DF score compared to children who were cooperative during the dental examination. Paryab and Hosseinbor previously described uncooperative behaviour as an indicator for DA, and high DA and bad past dental experience as important factors in predicting uncooperative behaviour in the dental clinic [14]. In addition, it was reported that while 27% of children with dental behaviour management problems (DBMP) have DF, 61% of fearful children have DBMP [37].

Our results also showed that children who felt pain during previous dental treatment/s, even if the level was low, had higher fear scores compared to others. This significant relationship between pain and DF was also confirmed recently [34].

The regression analysis in our study revealed that caries level was not associated with dental fear after controlling for confounders. This result is in agreement with previous studies that showed no relationship between dental caries and DF [30, 38, 39]. In contrast, other studies demonstrated that fearful children have more caries experience [4, 13, 15, 29].

This study assessed DF and the factors associated with it. However, the study has some limitations. One of these limitations was the inability to investigate the causal relationship of the factors with DF. Thus, our results do not provide definite information about the cause and effect relationships. Therefore, our results regarding the factors associated with DF must be treated with caution. Another limitation of the study is the fact that the assessment of behaviour during dental examination was carried out in schools. Assessing behaviour during a dental examination does not always identify the actual behaviour of the child during dental treatment. However, children who do not go to dentists because of DF are found within the school environment. In this study, 3.4% of the participants had not visited the dentist in the past year because of fear. In general, one of the limitations of questionnaire studies is the recall bias. However, to minimize recall bias in our study, the parent’s questionnaire was sent home with the children and the parents were given enough time to recall and answer the questionnaires. In addition, the questions were clear and asked about a recent period of time.

One strength of this study is that the children who participated completed the questionnaire independently in the school. Thus, it is known who provided the DF score data, which increases the validity of the results. This contrasts with different methodologies that allow the children to complete the questionnaire at home; thus, it is difficult to know who completed the questionnaire and so the validity is affected.

## Conclusion

DF is comparably low among 12–15 years old children in Jeddah, Saudi Arabia. There were significant relationships between DF and female sex, young age, going to public schools, visiting the dentist in a symptomatic pattern, avoidance of the dentist because of fear, feeling pain during previous dental visits, and poor behaviour during the dental examination. There were no significant relationships between DF and history of exposure to the dentist, visiting the dentist within the last year, caries experience, or type of treatment in previous dental visits. This study confirms the importance of visiting the dentist regularly, and the use of appropriate behavioural guidance and effective pain control during dental treatment to decrease the probability of DF. Evaluation of the DF level of the child before starting dental treatment, using an appropriate scale such as the CFSS-DS, may help the dentist to identify the behaviour of his/her patient and, therefore, choose suitable behavioural guidance.

## Additional file

Additional file 1: Parents’ Questionnaire. (DOCX 15 kb)

## Abbreviations

ANOVA: Analysis of variance
CFSS-DS: Children’s fear survey schedule-dental subscale
CPI: Community periodontal index
DA: Dental anxiety
DBMP: Dental behaviour management problems
DF: Dental fear
DFA: Dental fear and anxiety
DMFT: Decayed, missing, filled permanent teeth
WHO: World Health Organization
