# Supplementary material for: Effects of child characteristics and dental history on dental fear: cross-sectional study
Source: BMC Oral Health. 2018 Mar 7;18:33. doi: 10.1186/s12903-018-0496-4 (PMC5842627; doi:10.1186/s12903-018-0496-4)
Supplement: Supplementary file 1 — Parents’ Questionnaire. (DOCX 15 kb) [file 12903_2018_496_MOESM1_ESM.docx]

# **King Abdulaziz University Faculty of Dentistry**

**Department of Pediatric Dentistry**

**Parents’ Questionnaire**

Child name: ……………………………

Child age: …….….

Child grade: …………

Child gender: ………………………….

1. How often does your child go to the dentist?

a) Regularly every 6-12 months

b) On feeling pain only

c) Occasionally

d) He/she never went to a dentist

2. In the last year, has your child visited the dentist?

1. Yes
2. No

3. If no, why?

1. Financial reason(s)
2. No pain
3. No time
4. No need
5. Child fear

4. What procedures did his/her previous dental treatment include? (check all that apply):

1. Dental examination
2. Local anesthesia
3. Dental extraction
4. Tooth filling
5. Prophylaxis
6. Other(s)……………………………………..

5. During the previous dental visit, how was his/her behaviour?

1. Crying
2. Screaming
3. Resistant
4. Cooperative
5. Happy
6. I don’t know

7. During the previous dental visit, did he/she feel pain?

1. Yes
2. No
3. A little

**Thank you for taking the time to complete this survey!**
